# Mapping divided households and residency changes: the effect of couple separation on sexual behavior and risk of HIV infection

**DOI:** 10.1038/srep17598

**Published:** 2015-12-02

**Authors:** Laurence Palk, Sally Blower

**Affiliations:** 1 Center for Biomedical Modeling, Semel Institute of Neuroscience and Human Behavior, David Geffen School of Medicine, University of California, 760 Westwood Plaza, Office 27-423, Los Angeles, California 90095, USA

## Abstract

Using census data we identify geographic patterns in residency changes in Lesotho over a decade. Using kriging to spatially interpolate data from 8,510 households we identify regions where households have members temporarily living away from home (divided households). Further, using a multivariate analysis and data from 2,026 couples we determine whether a partners’ absence increases the likelihood of having extramarital partners and/or risk of HIV infection. Approximately 40% of individuals moved between 2001 and 2011; mainly to, and within, urbanized regions. Many households are divided: ~40% have members elsewhere in Lesotho, ~30% in South Africa (SA). Geographic patterns are apparent; they differ based on where the household member is living. Many couples were temporarily separated: ~50% of wives, ~20% of husbands. Separation was not a risk factor for HIV. Only men were more likely to have extramarital partners if their spouse was away: ~1.5 times if in Lesotho, ~3 times if in SA. The high degree of geographic mixing necessitates synchronizing interventions within Lesotho, and with SA, to successfully reduce transmission. It will be challenging to reduce concurrency in men with wives away from home. Our results are generalizable to other sub-Saharan countries where residency changes are common.

Changes in residency are a common occurrence in sub-Saharan African (SSA) countries with generalized HIV epidemics. Permanent and temporary changes occur, both within countries and across international borders^1^,^2^. However, there is little research on mapping geographic patterns of residency changes; and determining how many people move, and which areas they move between. Individuals who change residency, either on a permanent or temporary basis, often maintain links with their home communities; this linkage can connect localized HIV epidemics^3^. Previous research has focused on individuals who travel, finding an association between mobility and increased sexual behavior, and between mobility and risk of HIV infection^4^,^5^,^6^,^7^,^8^,^9^,^10^,^11^,^12^,^13^,^14^. The effect of couple separation on the partner who stays at home and does not travel has received little attention; specifically, it is not known whether a partners’ absence increases the likelihood of having extramarital partners and subsequently the risk of HIV infection.

We use Lesotho as an example of a SSA country with a generalized HIV epidemic; prevalence is ~20%^15^. Residency change is common; Lesotho and other countries in SSA have seen rapid urbanization since the 1980 s^16^. Many men and women in Lesotho live away from their families on a temporary basis, as they travel for employment as agricultural, domestic or mine workers. These individuals live for short periods either in different parts of the country or across the border in South Africa^17^,^18^. A map showing the location of cities and town in Lesotho and in nearby South Africa is given in Fig. 1. We use data from the 2011 Lesotho Demographic Survey and the 2009/10 Lesotho Demographic Health Survey to address three specific questions:

1. Which regions of the country have gained, and which regions have lost, residents over the past decade? Regions are considered in terms of healthcare districts (HCDs); Lesotho is divided into ten HCDs.
2. Which geographic regions have divided households? Divided households are defined as those with members who are temporarily living away from home.
3. Do individuals who have a spouse living away from home have a greater number of extramarital partners and/or risk of HIV infection than those with a spouse at home?

## Methods

Using the 2011 Lesotho Demographic Survey^19^ we estimated the number of individuals who moved between HCDs in the previous decade. We made these estimates by multiplying the population size of each HCD with the percentage that moved to every other HCD between 2001 and 2011. We used these results to map changes in residency.

We used data from the 2009/2010 Lesotho Demographic and Health Survey to determine which regions have divided households^20^. Data were collected from 9,391 households at 400 georeferenced cluster locations. For each household one member reported where they, and all other household members, were currently living: at home, elsewhere in Lesotho or in South Africa. We analyzed data from women 15 to 49 years old and men 15 to 59 years old, and excluded overnight guests. This reduced the sample to 10,615 men and 10,474 women from 8,510 households. Of this sample 3,075 men and 3,849 women completed an additional individual-level questionnaire (which provided data on sexual behavior) and were tested for HIV. At each cluster location (cluster locations are shown in Ref. [15]) we calculated the percentage of households who had adult members living away. We then mapped divided households using kriging. Kriging is a spatial interpolation technique that uses semi-variograms to model spatial dependency and to interpolate between cluster locations^21^. Our data has a high degree of spatial autocorrelation and hence it is particularly suited to this technique, although other methods would also have been appropriate^22^.

To determine whether the individuals who had a partner living away from home had more sex partners and/or were more likely to be infected with HIV we used data from both the household survey and the individual-level questionnaires. We used these data to identify 2,026 couples: 796 were identified because both partners completed the individual-level questionnaire and were tested for HIV. For these couples we have data for both partners on residency, sexual behavior and HIV status. We identified the remaining 1,230 couples by linking individuals who had completed the individual-level questionnaire and been tested for HIV (946 women and 284 men) with their partners through the household survey data. For these couples we have data for both partners on their residency, but data on sexual behavior and HIV status for only one of the partners.

We used the data from the 2,026 couples to conduct a gender-stratified multivariate analysis. HIV status and sexual behavior variables were used as response variables. The residency status of the partner was assessed as an explanatory variable. Odds ratios and confidence intervals were calculated adjusting for age and marital status.

## Results

A considerable proportion of the population changed their permanent residence between 2001 and 2011. We found ~15% of the population moved to a different HCD, and ~30% moved within their HCD. Figure 2 shows the direction of movements between HCDs. Movements range from ~1,000 individuals (blue data) to ~7,400 individuals (red data); two patterns are apparent, both related to urbanization. Individuals from the mountains and foothills in the southern and eastern HCDs moved to the four most urbanized HCDs (Mafeteng, Maseru, Berea and Leribe) in the lowlands of the northwest. Maseru HCD, which contains the capital city, received the greatest number of new residents (Fig. 2); residents came from every other HCD. There was also a great deal of movement within the urbanized regions in the lowlands (Fig. 2).

**Figure 1:**
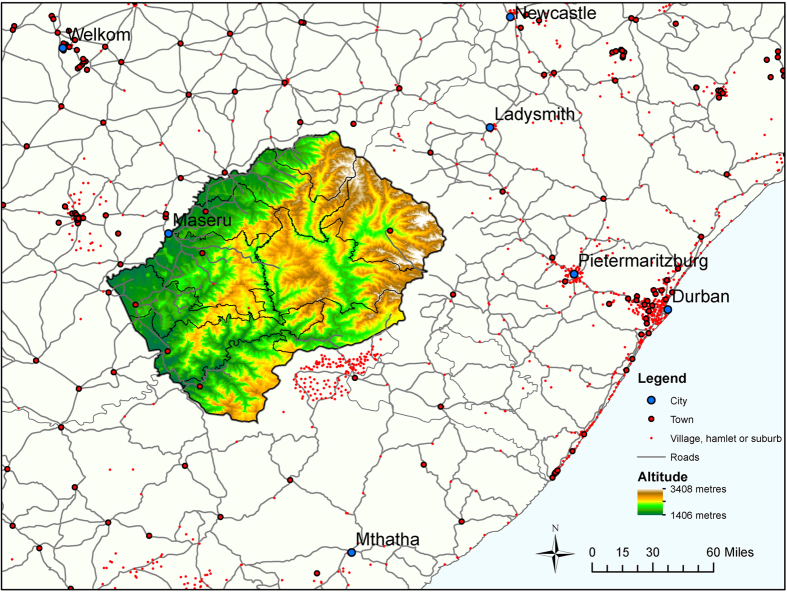
Map showing the geography of Lesotho and South Africa. Cities are shown as blue dots with labels. Towns, villages, hamlets and suburbs shown with red dots. Black lines show the boundaries of health care districts within Lesotho. Map created in ArcGIS using open source data^27^,^28^,^29^.

**Figure 2:**
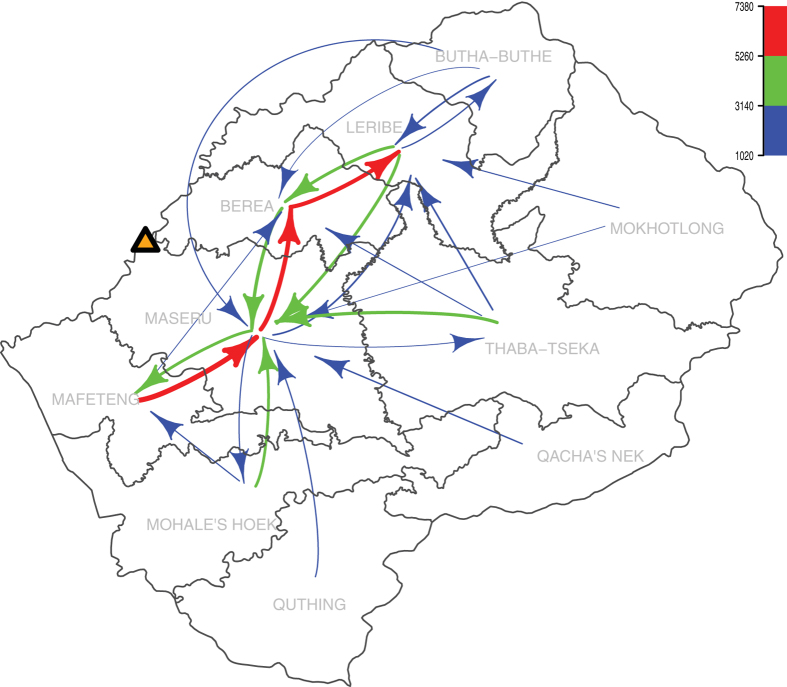
Map showing changes in permanent residency between 2001 and 2011. Arrows show the number of individuals moving from one HCD to another. Arrow width is representative of the number of individuals moving. Movements of less than 1000 individuals are not show. The yellow triangle shows the location of the capitol city, Maseru. The figure was produced using the R package ‘diagram’^30^,^31^.

**Figure 3:**
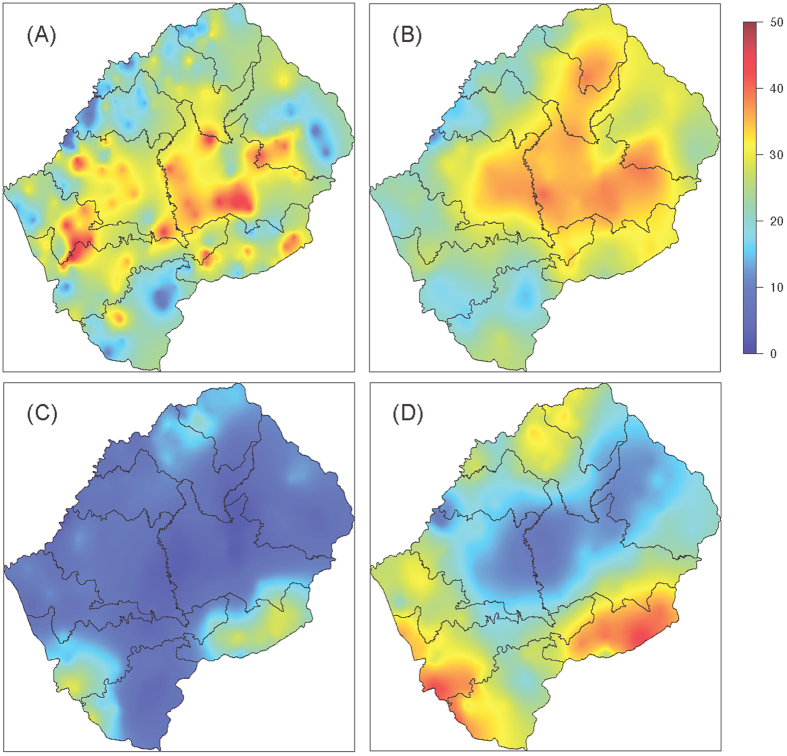
Maps showing the percentage of households with adult: (**A**) female and (**B**) male household members living elsewhere in Lesotho, and (**C**) female and (**D**) male household members living in South Africa. The maps were constructed using kriging, which was implemented using the R package geoR^30^,^32^.

Our results show there are a significant number of men and women who live away from home on a temporary basis. Countrywide, ~40% of households had a member living elsewhere in Lesotho, ~30% had members living in South Africa. Clear geographic patterns are evident in the maps shown in Fig. 3; the patterns differ based on whether the household member is elsewhere in Lesotho (Fig. 3A,B) or in South Africa (Fig. 3C,D). Almost half of households in the mountainous interior of the country, which has few employment opportunities, had family members living elsewhere in Lesotho (red data, Fig. 3A,B). In the more urbanized region, particularly around the capital Maseru (shown in Fig. 1), few households had members living elsewhere in Lesotho, ~10% (blue data, Fig. 3A,B). Households with members living in South Africa are concentrated in the border regions of the southwest, southeast and northeast of the country. In these regions almost half of households had male household members (red data, Fig. 3D) and ~10% to ~30% had female members living in South Africa (Fig. 3C).

Demographic statistics for individuals, stratified by their partners’ residency status, are shown in Table 1. Nearly all (~99%) of the couples in our analysis were married; women had a median age of 30 and men of 36. Many individuals reported their spouse was living temporarily away from home. Approximately 20% of husbands and ~15% of wives were elsewhere in Lesotho (Table 1); ~30% of husbands and ~5% of wives were in South Africa. HIV prevalence amongst the couples was greater than 20% in both genders. Men whose wives lived elsewhere in Lesotho had the highest prevalence, 31%. Both men and women reported having extramarital partners in the year before the survey: 7% to 9% of women and 24% to 44% of men. The highest percentage was in men whose wives were living in South Africa.

Results from the multivariate analysis are shown in Table 1. For both genders, having a spouse living away from home was not a risk factor for HIV infection. Women whose husband was living away from home were not more likely to report extramarital partners in the past year than those with their husband at home. However, a man whose wife was living elsewhere in Lesotho was ~1.5 times more likely to have had extramarital partners in the past year than a man whose wife was living at home (Table 1). Furthermore, a man whose wife was living in South Africa was ~3 times more likely to have had extramarital partners than a man whose wife was living at home (Table 1).

## Conclusions

Changes in residency in Lesotho, whether permanent or temporary, have the potential to increase HIV transmission and the difficulty of controlling the epidemic. We found there is a great deal of movement within Lesotho, both towards the most urbanized regions of the country and within these regions. HIV prevalence in Lesotho is greater in urban centers than in rural communities; consequently, urbanization is likely to intensify HIV transmission^15^,^22^. The high degree of geographic mixing that we have found in Lesotho links multiple communities: rural with rural, urban with urban, and rural with urban. Therefore it will be necessary to synchronize the introduction of interventions on a regional basis in order to successfully reduce transmission.

We found, in the border regions, Lesotho is tightly linked to South Africa. The mines in South Africa are an important source of employment for Lesotho men. Due to a high concentration of sex workers, HIV prevalence in the mines is particularly high^23^. There may be a high risk of cross border importation of HIV infections from South Africa into the specific areas on the border that we have identified. Our previous research found travel was associated with an increased risk of HIV infection for men who traveled frequently^14^. Therefore these regions may be particularly suitable for interventions that target migrant workers and discordant couples; notably, there is a high level of discordancy (14%) in Lesotho^24^. Such interventions are beginning to be implemented in Lesotho^25^,^26^. Further, our results imply that to effectively control the HIV epidemic in Lesotho will require close coordination with the South African Government.

Notably, we did not find an association - for married individuals who stay at home - between the residency status of their spouse and their risk of HIV infection. This may be due to a “saturation of risk”. Essentially all adults in Lesotho have a very high risk of acquiring HIV, this is due to the extremely high prevalence of HIV; in Lesotho 18% of women who have had only one lifetime partner are infected^15^.

Interestingly we found men, but not women, with a spouse living away from home were more likely to have extramarital partners in the previous year than those with their spouse living at home. Men whose wives were living in South Africa were the most likely to have extramarital partners. Unfortunately, we do not have data on the length of time that the wife was away from home. It is possible that wives living in South Africa were away from home for longer than wives who were living elsewhere in Lesotho. If so, this suggests that the longer a wife is away from home, the more likely a husband is to have extramarital partners. Since 24% of the married men in our sample were infected with HIV, men with extramarital partners are likely to be very important in the transmission of the virus. It is likely to be very challenging to find behavioral interventions that are effective in reducing concurrency in men with wives who are temporarily living away from home. Taken together, our results show that by using geographic mapping and considering changes in residency we can gain new insights into the dynamics of HIV epidemics. By considering the consequences of geographic mixing, it will be possible to develop more effective and targeted interventions.

## Additional Information

**How to cite this article**: Palk, L. and Blower, S. Mapping divided households and residency changes: the effect of couple separation on sexual behavior and risk of HIV infection. *Sci. Rep.* **5**, 17598; doi: 10.1038/srep17598 (2015).

## Figures and Tables

**Table 1:**
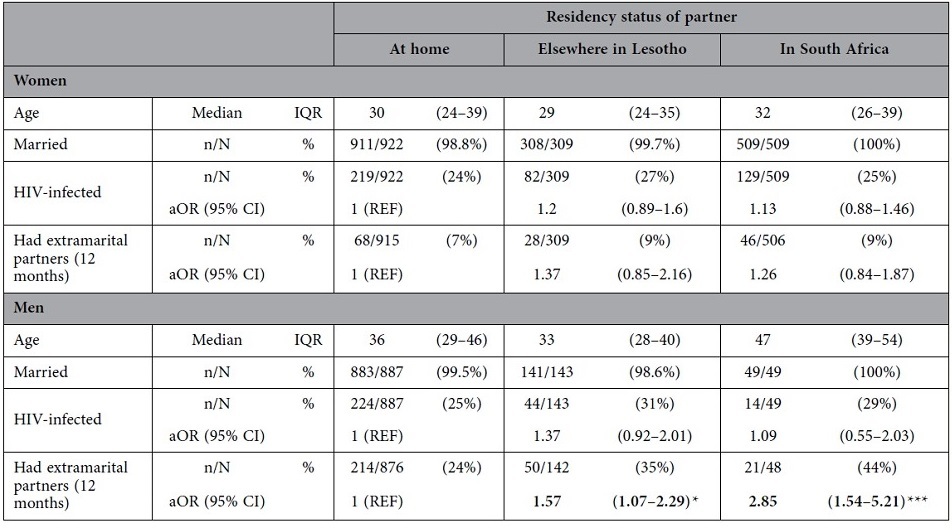
Summary statistics and adjusted odds ratios (aOR) for HIV infection and sexual risk behavior.

N denotes the sample size for the given gender and partners’ residency status. n is the number who have a given demographic characteristic, engage in a risk behavior or are HIV infected. IQR is the inter-quartile range and CI the confidence interval. Odds ratios are calculated adjusting for age and marital status. Asterisks denote the significance according to the following p-values: ***p < 0.001, **0.001 ≤ p < 0.01, *0.01 ≤ p < 0.05.
